# A learning health system approach to the COVID‐19 pandemic: System‐wide changes in clinical practice and 30‐day mortality among hospitalized patients

**DOI:** 10.1002/lrh2.10304

**Published:** 2022-01-27

**Authors:** Erin K. McCreary, Kevin E. Kip, J. Ryan Bariola, Mark Schmidhofer, Tami Minnier, Katelyn Mayak, Debbie Albin, Jessica Daley, Kelsey Linstrum, Erik Hernandez, Rachel Sackrowitz, Kailey Hughes, Christopher Horvat, Graham M. Snyder, Bryan J. McVerry, Donald M. Yealy, David T. Huang, Derek C. Angus, Oscar C. Marroquin

**Affiliations:** ^1^ Division of Infectious Diseases, Department of Medicine University of Pittsburgh School of Medicine Pittsburgh Pennsylvania USA; ^2^ Health Services Division Clinical Analytics, UPMC Pittsburgh Pennsylvania USA; ^3^ Division of Cardiology, Dept of Medicine University of Pittsburgh School of Medicine Pittsburgh Pennsylvania USA; ^4^ Health Services Division UPMC Wolff Center and Quality Offices, UPMC Pittsburgh Pennsylvania USA; ^5^ Media Relations Department UPMC Communications, UPMC Pittsburgh Pennsylvania USA; ^6^ UPMC Enterprises UPMC Supply Chain Management/HC Pharmacy, UPMC Pittsburgh Pennsylvania USA; ^7^ UPMC Health System UPMC Office of Healthcare Innovation Pittsburgh Pennsylvania USA; ^8^ Department of Pharmacy UPMC Pinnacle, UPMC Pittsburgh Pennsylvania USA; ^9^ Department of Critical Care Medicine University of Pittsburgh School of Medicine Pittsburgh Pennsylvania USA; ^10^ Department of Critical Care Medicine UPMC Children’s Hospital of Pittsburgh Pittsburgh Pennsylvania USA; ^11^ Department of Medicine, Division of Pulmonary Allergy, and Critical Care Medicine Pittsburgh Pennsylvania USA; ^12^ Department of Emergency Medicine University of Pittsburgh School of Medicine Pittsburgh Pennsylvania USA

**Keywords:** dexamethasone, regulatory guidelines, remdesivir, scientific dissemination, temporal trends, tocilizumab

## Abstract

**Introduction:**

Rapid, continuous implementation of credible scientific findings and regulatory approvals is often slow in large, diverse health systems. The coronavirus disease 2019 (COVID‐19) pandemic created a new threat to this common “slow to learn and adapt” model in healthcare. We describe how the University of Pittsburgh Medical Center (UPMC) committed to a rapid learning health system (LHS) model to respond to the COVID‐19 pandemic.

**Methods:**

A treatment cohort study was conducted among 11 429 hospitalized patients (pediatric/adult) from 22 hospitals (PA, NY) with a primary diagnosis of COVID‐19 infection (March 19, 2020 ‐ June 6, 2021). Sociodemographic and clinical data were captured from UPMC electronic medical record (EMR) systems. Patients were grouped into four time‐defined patient “waves” based on nadir of daily hospital admissions, with wave 3 (September 20, 2020 ‐ March 10, 2021) split at its zenith due to high volume with steep acceleration and deceleration. Outcomes included changes in clinical practice (eg, use of corticosteroids, antivirals, and other therapies) in relation to timing of internal system analyses, scientific publications, and regulatory approvals, along with 30‐day rate of mortality over time.

**Results:**

The mean (SD) daily number of admissions across hospitals was 26 (29) with a maximum 7‐day moving average of 107 patients. System‐wide implementation of the use of dexamethasone, remdesivir, and tocilizumab occurred within days of release of corresponding seminal publications and regulatory actions. After adjustment for differences in patient clinical profiles over time, each month of hospital admission was associated with an estimated 5% lower odds of 30‐day mortality (adjusted odds ratio [OR] = 0.95, 95% confidence interval: 0.93‐0.97, *P* < .001).

**Conclusions:**

In our large LHS, near real‐time changes in clinical management of COVID‐19 patients happened promptly as scientific publications and regulatory approvals occurred throughout the pandemic. Alongside these changes, patients with COVID‐19 experienced lower adjusted 30‐day mortality following hospital admission over time.

## 1 INTRODUCTION

Integration of evidence‐based practices is notoriously slow, especially at larger, diverse, health care systems. The emergence of a rapidly spreading, severe respiratory virus pandemic created a heightened need for change in this common approach.^1^, ^2^ Current research infrastructure and information technology systems facilitate unprecedented volume and speed of pandemic‐related information, and data sharing in the biomedical literature, social media, and other resources allows insights to flow much more quickly.^3^ Making efficient, optimal use of this massive, constantly changing information is paramount to minimize the deadly impact of the coronavirus disease 2019 (COVID‐19) pandemic and for the health and welfare of humanity at large.

In addition to the need for coordinated global approaches to pandemics,^1^ individual health care delivery systems must seek to give equitable, evidence‐based care across institutions regardless of geographical region or hospital type.^4^ In this realm, a *learning health system* (LHS) is an ideal organizing principle to inform evidence‐based responses to public health emergencies like COVID‐19.^4^ The LHS concept is characterized as an environment in which “science, informatics, incentives, and culture are aligned for continuous improvement and innovation, with best practices seamlessly embedded in the delivery process and new knowledge captured as an integral by‐product of the delivery experience.”^5^

Seeking to embrace the LHS model, the UPMC health system leveraged its science, data, and analytics capabilities and established the multidisciplinary COVID‐19 Therapeutics Committee in early 2020. The purpose of this Committee was to evaluate any possible COVID‐19 treatment option and rapidly disseminate updated guidelines to all institutions within the system. The Committee also coordinated with information technology specialists to build forcing functions into several electronic medical records (EMRs) to enforce practice guideline recommendations and also collaborated with research teams to integrate clinical practice with clinical trial enrollment across the enterprise. This LHS process, coupled with regular internal COVID‐19 analyses from the UPMC Clinical Analytics Team (described in Methods), formed the basis for establishing, disseminating, and documenting data‐driven clinical recommendations to all UPMC outpatient and in‐patient facilities caring for patients with COVID‐19.

We describe the UPMC LHS approach to the COVID‐19 pandemic since March 2020. We share processes on the development and dissemination of clinical guidelines that occurred in a near real‐time manner across the entire UPMC system. We also share quantitative results of how such changes mirrored credible findings and information from key scientific publications and regulatory approvals. This is followed by temporal assessment of the 30‐day rate of mortality of hospitalized patients with COVID‐19.

## 2 QUESTION OF INTEREST

How have hospital patient volumes, patient clinical management, and 30‐day mortality changed since the onset of the COVID‐19 pandemic within a large, multihospital LHS?

## 3 METHODS

### 3.1 Influence of UPMC COVID‐19 Therapeutics Committee

The UPMC COVID‐19 Therapeutics Committee was appointed by health system senior leadership in March 2020. This Committee was a subcommittee of the System Pharmacy and Therapeutics Committee (P&T); however, it was given ability to vote and implement therapeutics guideline changes in real‐time rather than going through traditional P&T pathways. The Committee met weekly at first and then biweekly. Membership included physicians, pharmacists, hospital leadership, and other stakeholders from academic and community hospitals with clinical, operational, and research experience. A pharmacist and two physicians co‐chair the Committee. An internal communications representative attended all meetings and worked, in real time, to update the system intranet as relevant and help draft system‐wide communications for clinicians. Information technology specialists also served as Committee liaisons. Finally, an intensive care unit (ICU) service center collaborated with the Committee and provided system‐level recommendations, ICU surge provider staffing algorithms, and tele‐medicine support for patients throughout the system to limit hospital transfers.^6^

We evaluated the influence of the UPMC COVID‐19 Therapeutics Committee on change in COVID‐19 clinical practice by time series plotting of the prevalence of in‐hospital use of selected medications in relation to internal analyses and key scientific publications and regulatory approvals routinely reviewed by the committee. Regulatory approvals included things such as Emergency Use Authorizations and state or national mandates for provision of care (ie, restrictions on outpatient hydroxychloroquine prescriptions). Consistent with the “rapid‐learning health system” described by Greene et al,^7^ the UPMC COVID‐19 Therapeutics Committee charge was to continuously evaluate evidence to create and disseminate treatment recommendations across the UPMC system. The process included: (a) weekly to biweekly review of internal analyses of COVID‐19 patient testing, clinical practice, and outcomes generated from the CDW; (b) interim review of results from UPMC‐led Randomized Embedded Multifactorial Adaptive Platform for COVID‐19 (REMAP‐COVID) trials, a global adaptive platform with response‐adaptive randomization for trials of hospitalized and ambulatory patients with COVID‐19;^8^, ^9^ (c) weekly and ad‐hoc review of key external scientific publications, press releases, and regulatory approvals of COVID‐19 treatment approaches; (d) consensus determination of patient criteria and clinical instructions for use (and nonuse) of established and emerging treatment approaches, including consideration to drug shortages and prioritization patients for use in settings of shortage; (e) creation of EMR‐embedded forcing functions to enforce therapeutics recommendations and guide prescribing at the point of care; (f) empowerment of local pharmacists to review and approve all COVID‐19‐related medications within the context of the guidelines; and (g) system‐wide dissemination of continuously updated treatment recommendations using multimodal media sources. Whenever a change was made (eg, guideline update, new EHR‐based forcing function implemented), it was made live, and education was disseminated on the same date, in the same manner, at every site (academic and community).

The system‐wide dissemination of treatment guidelines to all physicians and other clinicians affiliated with UPMC occurred through email notifications, computer screensavers, educational webinars, and formal directives from the chair of the Committee. A COVID‐19 therapeutics webpage was built into the system intranet. The COVID‐19 Therapeutics Committee also created continuous, updated recommendations on the use of monoclonal antibodies for ambulatory COVID‐19 patients beginning in November 2020; however, the present analysis is restricted to treatment of hospitalized patients and omits that intervention.

### 3.2 Patient Population

Within this LHS, there were 5 large, academic hospitals (2474 licensed beds), 8 large, community hospitals (2293 licensed beds), and 9 small, community hospitals (1107 licensed beds) (Table S1). We identified 323,101 patients (pediatric and adult) with nucleic acid amplification tests for SARS‐CoV‐2 performed at a UPMC facility during the period March 17, 2020, to June 6, 2021. Of 53,183 patients (16.5%) testing positive, 9554 (18.0%) were hospitalized at one of 22 UPMC hospitals. An additional 1875 COVID‐19 patients were hospitalized at a UPMC hospital with testing performed outside the UPMC system, resulting in a total of 11 429 hospitalized patients for analysis (Figure S1 **).**

### 3.3 Sources of data

We used data captured in the EMR and ancillary clinical systems, all of which are aggregated and harmonized in a Clinical Data Warehouse (CDW). UPMC is a 40‐hospital integrated academic healthcare system providing care principally within central and western Pennsylvania (USA). For the 22 hospitals with complete EMR data in the CDW, we accessed all key clinical data, including detailed sociodemographic and medical history data, diagnostic and clinical tests conducted, surgical and other treatment procedures performed, prescriptions ordered, and billing charges on all outpatient and in‐hospital encounters, with diagnoses and procedures coded based on the International Classification of Diseases, Ninth and Tenth revisions (ICD‐9 and ICD‐10, respectively).

### 3.4 Outcomes

We assessed changes in utilization of COVID‐19 pharmacotherapy, level of oxygen support during hospitalization, and 30‐day mortality from the index date of hospital admission. Pharmacotherapy and oxygen support were determined by the presence of charge codes within UPMC billing software. We assessed 30‐day mortality by the hospital discharge disposition of “Ceased to Breathe” sourced from the inpatient Medical Record System, as well as deaths after discharge identified with the Death Master File (DMF) from the Social Security Administration (SSA) (NTIS 2021) as an external data source.

### 3.5 Explanatory variables

For assessment of temporal changes, we categorized the study analysis period into 4 discrete “waves” based on empirical change in hospital admissions within the UPMC system. We chose the 4‐wave classification scheme (Figure S2) based on the start and nadir of individual waves. However, because Wave 3 (September 29, 2020 ‐ March 10, 2021) had dramatically higher hospital admissions and discharges, we split this wave at its zenith to assess its impact during rapid acceleration and deceleration. For assessment of variation between waves, we considered demographic variables, clinical history and medical comorbidities, laboratory values, vital signs, and medication use, with a focus on indicators of changing clinical practice such as use and timing of specific medications. We further assessed changes in COVID‐19 clinical practice by the date of important scientific and regulatory events, as formally reviewed by the UPMC COVID‐19 Therapeutics Committee. We also assessed potential variation in clinical practice across the 22 hospitals by classification as “large academic” (*n* = 5), “large community” (*n* = 8), or “small community” (*n* = 9) (Table S1).

### 3.6 Statistical methods

We describe changes over time in COVID‐19 hospital admissions using 7‐day moving mean and median values. We plotted temporal changes in pharmacotherapy used in‐hospital on a weekly basis and anchored to important scientific and regulatory events. Medication use and oxygen support (proportion of patients) plots by wave of hospital admission were evaluated by the Cochran‐Mantel‐Haenszel test of trend. We compared presenting characteristics of hospitalized patients across the 4 waves using analysis of variance (ANOVA) or Wilcoxon tests for continuous variables (based on distributional properties) and chi‐square tests for categorical variables. Crude rates of 30‐day mortality for test positive and hospitalized patients by wave were censored at May 7, 2021 (ie, to allow 30‐day follow‐up for all patients). A general linear model specifying the binomial distribution and logit link, and including site (hospital) as a random effect, was fit using 30‐day mortality as the dependent variable. Stepwise selection of pretreatment explanatory variables was initially determined with the use of logistic regression analysis. Date of hospital admission was added to the model at the last stage to assess whether the odds of 30‐day mortality changed over time after adjustment for different patient characteristics. We did not impute missing values in any analyses. A two‐sided type I error rate of 0.05 was used, and all analyses were conducted using the SAS System, Version 9.4 (SAS Institute, Cary, NC). We used The REporting of studies Conducted using Observational Routinely‐collected health Data (RECORD) approach^10^ (see Table S2). Our study received formal ethics approval by the UPMC Ethics and Quality Improvement Review Committee (Project ID Project ID 2882).

## 4 RESULTS

### 4.1 Hospital admissions

Over the 14+ month study period, the mean (SD) daily number of admissions across all hospitals was 26 (29) with median of 17, IQR of 6‐31, maximum 7‐day moving mean of 107, and steep acceleration and deceleration during wave 3 from late September 2020 to early March 2021 (Figure S2). The mean hospital admission rate per day by wave was 4.0 (wave 1), 9.1 (wave 2), 46.1 (wave 3a), 48.0 (wave 3b), and 24.0 (wave 4).

### 4.2 Temporal changes in clinical practice

**FIGURE 1:**
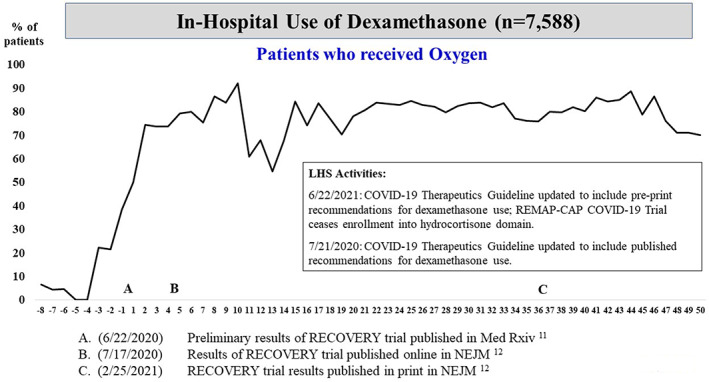
Plot of weekly prevalence (%) of in‐hospital use of dexamethasone among patients who received oxygen. On the x‐axis, negative numbers reflect weeks prior to seminal event “A,” the date (June 22, 2020) in which preliminary results of the RECOVERY trial were published in *Med Rxiv*. Positive numbers reflect weeks after seminal event A

**FIGURE 2:**
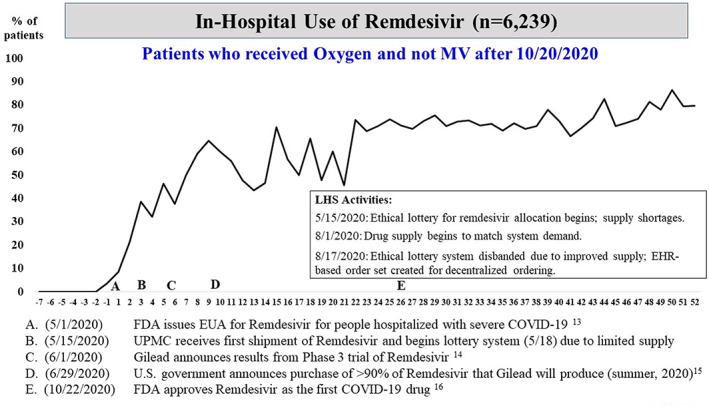
Plot of weekly prevalence (%) of in‐hospital use of remdesivir among patients who received oxygen (but not mechanical ventilation after October 20, 2020). On the x‐axis, negative numbers reflect weeks prior to seminal event “A,” the date (May 1, 2020) in which the Food and Drug Administration (FDA) issued Emergency Use Authorization (EUA) for remdesivir for patients hospitalized with severe coronavirus disease 2019 (COVID‐19)

The COVID‐19 Therapeutics Committee published 45 iterations of the clinical practice guideline during the study period. Among patients who received any form of supplemental oxygen, there was rapid system‐wide implementation in the use of dexamethasone immediately around the date in which initial positive results of the RECOVERY trial were published as a preprint^11^ (Figure 1). Of note, subsequent peer‐review publication^12^ did not trigger an added uptake in the use of dexamethasone. A steep increase in the use of remdesivir among patients on oxygen therapy occurred after Emergency Use Authorization (EUA) granted by the FDA^13^ and subsequent public announcements and regulatory actions^14^, ^15^, ^16^ (Figure 2). There was no appreciable variation in the use of dexamethasone or remdesivir by volume or type across the 22 UPMC hospitals (Figures S3 and S4). Remdesivir was allocated via an ethical lottery system from May 15 through August 1, 2020, during times drug supply was scarce.^17^

**FIGURE 3:**
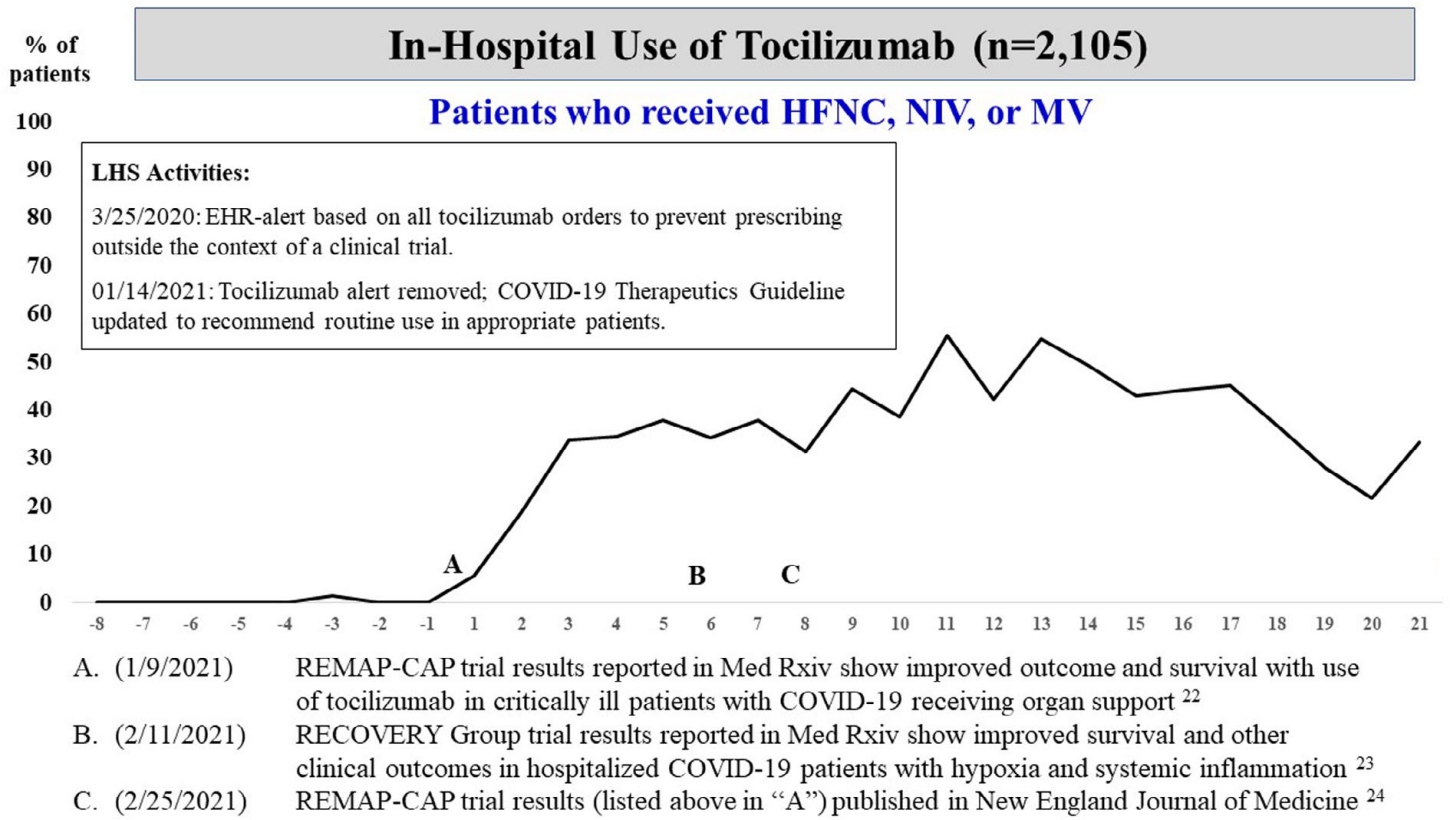
Plot of weekly prevalence (%) of in‐hospital use of tocilizumab among patients who received high‐flow nasal cannula (HFNC), BiPAP/CPAP (NIV), or mechanical ventilation (MV). On the x‐axis, negative numbers reflect weeks prior to seminal event “A,” the date (January 9, 2021) in which tocilizumab trial results were published among critically ill patients with coronavirus disease 2019 (COVID‐19) who were receiving organ support

In contrast, despite widespread publicity,^18^, ^19^ our group recommended no role for hydroxychloroquine outside of the context of a clinical trial. Subsequently, in‐hospital use was very low (<6%) and did not vary over time, including after FDA EUA revocation of hydroxychloroquine,^20^, ^21^ and thus may be represented solely by patients taking this medication for a non‐COVID‐19 indication or enrolled in a clinical trial (Figure S5). More recently, an increase in the use of tocilizumab among eligible patients occurred immediately following preprint release of the REMAP‐CAP trial results^22^ (Figure 3) and a few weeks after publication of the RECOVERY^23^ and REMAP‐CAP trial results.^24^

Among patients who received oxygen therapy, in‐hospital use of corticosteroids was 80% or higher starting in wave 2. Two‐thirds or more of all patients received steroids within 1 day of admission. Beginning in wave 3, about three‐quarters of all clinically appropriate patients received remdesivir (almost always within 1 day of admission) (Figure S6). Use of noninvasive ventilation did not vary appreciably across waves, whereas use of mechanical ventilation was markedly lower after wave 1 (Figure S6).

### 4.3 Temporal changes in patient characteristics and 30‐day mortality

Hospitalized patients in waves 1 and 3a/3b were significantly older than patients in wave 2 (about 3‐4 years), and the most recent wave 4 patients were the youngest with mean (median) age of 59.5 (62) years and about a quarter (27%) being age 50 years or younger (Table S3). In aggregate, patients in waves 1 and 3 generally presented with more comorbidities, higher estimated 90‐day probability of mortality, and higher neutrophil to lymphocyte ratio (NLR) and systemic inflammatory index than patients in waves 2 and 4 (Table S3).

Among all test positive patients (hospitalized and not hospitalized), the 30‐day mortality rate ranged from a high of 5.6% in wave 1 to a low of 2.2% in wave 4 (Table 1). Similar in direction, among hospitalized patients, the 30‐day mortality rate ranged from a high of 19.8% in wave 1 to a low of 8.5% in wave 4. Consistent with different risk profiles across the 4 waves, 30‐day mortality was highest in wave 1, intermediate in wave 3, and lowest in waves 2 and 4. After statistical adjustment, factors independently associated with 30‐day mortality rate after hospitalization included older age (16% increased odds per 5 years), male gender (24% increased odds), estimated risk of mortality within 90 days after being hospitalized (11% increased odds per 5 percentage points), and higher white blood cell, alanine aminotransferase (ALT), and nod‐like receptors (NLRs) (Table 2). Of note, adjusting for different risk profiles, each month of hospital admission to the UPMC system was associated with an estimated 5% lower odds of 30‐day mortality (adjusted OR = 0.95, 95% confidence interval: 0.93‐0.97, *P* < .001). In stratified analyses, adjusted odds ratios for hospitals classified as large academic, large community, and small community were 0.95, 0.94, and 0.97, respectively.

**TABLE 1:** Thirty‐day mortality rate of tested positive and hospitalized cases by wave

Wave	Patient time period	All test positives		Hospitalized cases	
		N	Rate (%)	N	Rate (%)
1	March 19 – June 16, 2020	1369	5.6	358	19.8
2	June 17 – September 19, 2020	3926	2.5	859	10.1
3a	September 20 – December 13, 2020	21 471	2.8	3925	14.9
3b	December 14, 2020 – March 10, 2021	18 637	2.9	4174	13.0
4	March 11 – May 7, 2021	6378	2.2	1674	8.5

**TABLE 2:** Odds ratios of factors associated with 30‐day mortality (n = 10 763 hospitalized patients)

Factor	Unadjusted OR	Adjusted OR	95% CI	*P*‐value
Age (per 5 years)	1.25	1.16	1.13‐1.20	<.001
Male gender	1.41	1.24	1.06‐1.45	.008
Estimated risk of mortality within 90‐days after being hospitalized (per 5 percentage points)^a^	1.17	1.11	1.09‐1.14	<.001
Log white blood cell count at hospital admission	2.02	1.41	1.25‐1.58	<.001
Log alanine aminotransferase (ALT) at hospital admission	1.25	1.41	1.29‐1.55	<.001
Neutrophil to lymphocyte ratio (per quintile)	1.41	1.28	1.21‐1.35	<.001
Date of hospital admission (per month)	0.95	0.95	0.93–0.97	<.001

*Note*: Odds ratios were calculated from a general linear model specifying the binomial distribution and logit link and including hospital as a random effect.

^a^ Risk score is derived from an internally validated algorithm that is comprised of a range of variables predictive of mortality including socio‐demographics, medical history, recent laboratory values, and prior health care utilization.

## 5 DISCUSSION

In 2009, the National Academy of Medicine (NAM) called for development of an LHS, setting a goal that by 2020, “…90 percent of clinical decisions will be supported by accurate, timely, and up‐to‐date clinical information, and will reflect the best available evidence.”^25^ The importance of this LHS goal is emblematic with the COVID‐19 pandemic.^26^ While lacking the ability to demonstrate cause and effect, the fact that the adjusted risk of in‐hospital mortality among hospitalized COVID‐19 patients at UPMC hospitals has decreased monthly by an average of 5% suggests a consistent learning effect to improved patient care. The 30‐day mortality rates and general trend over time (ie, reduction in mortality risk) from our institution are consistent with in‐hospital mortality results reported from the National Center for Health Statistics^27^ and a large cohort study of 209 US acute care hospitals of variable size in urban and rural areas.^28^ Additionally, within our study, there was no appreciable variation in type or volume of pharmacotherapy agents utilized for patients with COVID‐19 across 22 hospitals, achieving the goal of equity and access regardless of patient zip code. Importantly, this model continues and can rapidly adapt as needed for SARS‐CoV‐2 variants, vaccination efforts, and other key variables.

In addition to continuous evaluation of UPMC internal analyses and controlled clinical trials, the COVID‐19 Therapeutics Committee has evaluated the surge of COVID‐19‐related preprints and peer‐reviewed publications that have emanated on an unprecedented scale throughout the pandemic.^29^, ^30^, ^31^ This placed a premium on expertise in evaluating the merits of published information. While our committee recognized the benefits of and thus implemented steroids, remdesivir, and *tocilizumab* in selected COVID‐19 patients, it refuted use of hydroxychloroquine despite its EUA, given the existing data.^32^

The time between clinical evidence arising and uniform implementation of use was in days‐to‐weeks, rather than months‐to‐years, which has been the traditional gap for implementation of findings from RCTs into clinical practice.^33^, ^34^, ^35^ We invested substantial efforts in the use of near‐real time data and evidence (as per NAM LHS guidance), especially when the lack of available therapies fueled adoption of both warranted and unwarranted treatments. The average monthly risk‐adjusted decrease in mortality of 5% observed in our healthcare system is noteworthy given the overall worse clinical profile of patients seen in wave 3. While utilization of pharmacotherapy is the focus of this analysis, it is likely that the observed improvements are multifactorial in nature. Alongside the Therapeutics Committee, an ICU management group made real‐time recommendations surrounding respiratory support strategies and other critical, supportive care, and a system‐wide infection prevention taskforce guided testing, tracing, isolation, and use of personal protective equipment. Accordingly, we posit there were changes of unmeasured practice patterns (ie, ventilation strategies) learned over time that also contributed to the improved outcomes, and the rapid implementation of approved pharmacotherapies is a surrogate marker of system‐wide learning. Lastly, while improvements in outcomes over time are natural to the progression of science and medical practice, the fact that the improvement seen in our healthcare system happened in a short time and mostly prior to mass vaccination, speaks, at least in part, to the importance of our system’s embracing the organizational push to be an LHS.

While desirable, no formal criteria or certification process exists for an institution to be designated as an LHS.^4^ One component we believe is essential is embedding of randomized controlled trial procedures into routine care processes using existing institutional infrastructure and electronic health records.^8^ This approach defines broad eligibility criteria and aims to enroll as many “real‐world” patients as possible to continuously evaluate therapies believed to be potentially efficacious. The key is avoiding “research” and “care” schisms, but rather use all care as an opportunity to learn about care improvement. Randomization is an added tool for some efforts, allowing adaptation as the trial evolves such that subjects are preferentially randomized to receive better performing arms based on interim analyses—termed “response adaptive randomization.”^36^ This was accomplished at our hospitals by embedding REMAP‐CAP enrollment into the EMR, screening all patients with COVID‐19 at all hospitals for trial eligibility, and integrating trial enrollment with Therapeutics Committee treatment guidelines.^37^ Similarly, when treatment resources are limited and equitable lottery systems are implemented (eg, Remdesivir), this “natural experiment” can be analyzed against nontreated controls.

There are some limitations to our study; because this is the experience in one, albeit large, integrated healthcare system in Western Pennsylvania, the generalizability of our findings may be questioned. However, the fact that we saw similar findings across our different sites suggests that our findings are applicable across academic, community, and rural hospitals. In addition, we cannot determine the extent to which the therapeutic interventions implemented uniformly by the UPMC COVID‐19 Therapeutics Committee contributed to lower adjusted mortality over time, as opposed to other less well‐documented clinical practices that may have been implemented over time (ie, mechanical ventilation). Moreover, we cannot directly compare our lower adjusted mortality risk over time to similar findings that have been reported among studies with more hospitals and wider geographic distribution.^27^, ^28^ The LHS description and results presented herein are not meant to be content‐ or institution‐specific, but rather to illustrate some of the processes that can be used to support the NAM imperative for clinical decisions that are supported by accurate, timely, and up‐to‐date clinical information that reflects the best available evidence.^25^ On a broader level, we support the stated advocacy for a learning health network that promotes collaboration among health systems, community‐based organizations, and government agencies, especially during public health emergencies.^4^

## 6 CONCLUSION

Other institutions have qualitatively described their respective LHS processes employed in response to the COVID‐19 pandemic,^38^, ^39^ with limited quantitative temporal assessment of clinical outcomes.^40^ We believe our analysis and description is the first to empirically document how COVID‐specific processes employed within an LHS were actually implemented to achieve timely changes in clinical practice on a system level. We recommend that institutions in describing their respective LHS do so by linking (and presenting) processes and sources of information that were used in the establishment and dissemination of clinical care guidelines with data‐documented temporal changes in clinical practice and patient outcomes.

## CONFLICT OF INTEREST

None of the authors received any payments or influence from a third‐party source for the work presented, and none report any potential conflicts of interest.

## Supporting information


**Table S1** Listing of the University of Pittsburgh Medical Center (UPMC) hospitals by type, bed capacity, and volume
**Table S2**. Checklist: The REporting of studies Conducted using Observational Routinely‐Collected health Data (RECORD) statement.
**Table S3**. Presenting characteristics of hospitalized patients by admission wave

**Figure S1** Diagram of severe acute respiratory syndrome coronavirus 2 (SARS‐CoV‐2) testing performed within the University of Pittsburgh Medical Center (UPMC) system, including COVID‐19 hospitalized patients aggregated from patients tested within and outside of the UPMC system.

**Figure S2** Plot of 7‐day moving average of coronavirus disease 2019 (COVID‐19) hospital admissions by empirically defined “waves” based on nadir and zenith (wave 3) of admissions. The time periods for the waves were as follows: Wave 1: ‐ March 19 ‐ June 16, 2020; wave 2: June 17 ‐ September 19, 2020; wave 3a: September 20‐December 13, 2020; wave 3b: December 14, 2020 ‐ March 10, 2021 and wave 4: March 11 ‐ June 6, 2021.

**Figure S3** Plot of 4‐week prevalence (%) of in‐hospital use of dexamethasone among patients who received oxygen by hospital classification. On the x‐axis, negative numbers reflect weeks prior to seminal event “A”, the date (June 22, 2020) in which preliminary results of the RECOVERY trial were published in *Med Rxiv*. Positive numbers reflect weeks after seminal event A.

**Figure S4** Plot of 4‐week prevalence (%) of in‐hospital use of remdesivir among patients who received oxygen (but not mechanical ventilation after October 20, 2020) by hospital classification. On the x‐axis, negative numbers reflect weeks prior to seminal event “A,” the date (May 1, 2020) in which the Food and Drug Administration (FDA) issued Emergency Use Authorization (EUA) for remdesivir for patients hospitalized with severe coronavirus disease 2019 (COVID‐19).

**Figure S5** Plot of weekly prevalence (%) of in‐hospital use of hydroxychloroquine. On the x‐axis, negative numbers reflect weeks prior to seminal event “A,” the date (March 28, 2020) in which the Food and Drug Administration (FDA) granted EUA of hydroxychloroquine for coronavirus disease 2019 (COVID‐19) patients.

**Figure S6** Stacked bar charts (100%) of the percentage of hospitalized patients treated with steroids (upper left), remdesivir (upper right), bilevel positive airway pressure (BiPAP)/continuous positive airway pressure (CPAP) (lower left), and mechanical ventilation (lower right) by wave of hospital admission. For steroids, the denominator is patients on oxygen therapy. For remdesivir, the denominator is patients on oxygen therapy and not mechanical ventilation after October 20, 2020. Light shading: treatment not used; intermediate shading: treated provided within 1 day of hospital admission; darker shading: treatment provided after the first day of hospital admission. P‐values are based on the Cochran‐Mantel‐Haenszel test of trend.
