# Supplementary material for: A learning health system approach to the COVID‐19 pandemic: System‐wide changes in clinical practice and 30‐day mortality among hospitalized patients
Source: Learn Health Syst. 2022 Jan 27;6(3):e10304. doi: 10.1002/lrh2.10304 (PMC9284933; doi:10.1002/lrh2.10304)
Supplement: Supplementary file 1 — Table S1 Listing of the University of Pittsburgh Medical Center (UPMC) hospitals by type, bed capacity, and volume Table S2. Checklist: The REporting of studies Conducted using Observational Routinely‐Collected health Data (RECORD) statement. Table S3. Presenting characteristics of hospitalized patients by admission wave [file LRH2-6-e10304-s005.docx]

**Supplemental Table 1. Listing of UPMC Hospitals by Type, Bed Capacity, and Volume**

| **Hospital Type** | **Hospital Name** | **Number of**  **Licensed Beds** | **Number of Patients**  **in Analysis** |
| --- | --- | --- | --- |
| Large Academic | Children’s | 315 | 230 |
| Large Academic | Magee | 335 | 379 |
| Large Academic | Mercy | 404 | 857 |
| Large Academic | Presbyterian | 900 | 959 |
| Large Academic | Shadyside | 520 | 936 |
| Large Community | Altoona | 380 | 1444 |
| Large Community | East | 155 | 751 |
| Large Community | Hamot | 412 | 849 |
| Large Community | McKeesport | 216 | 442 |
| Large Community | Passavant | 394 | 970 |
| Large Community | St. Margaret | 249 | 719 |
| Large Community | Williamsport | 224 | 866 |
| Large Community | Western Psych | 263 | 77 |
| Small Community | Bedford | 49 | 196 |
| Small Community | Chautauqua | 277 | 246 |
| Small Community | Cranberry | 35 | 89 |
| Small Community | Horizon | 106 | 262 |
| Small Community | Jameson | 236 | 408 |
| Small Community | Lock Haven | 25 | 118 |
| Small Community | Muncy | 156 | 74 |
| Small Community | Northwest | 126 | 359 |
| Small Community | Soldiers & Sailors | 67 | 198 |

**Supplemental Table S2. Checklist: The REporting of studies Conducted using Observational Routinely-Collected health Data (RECORD) statement.**

|  | **Item No.** | **STROBE items** | **Location in manuscript where items are reported** | **RECORD items** | **Location in manuscript where items are reported** |
| --- | --- | --- | --- | --- | --- |
| **Title and abstract** | | | | | |
|  | 1 | (a) Indicate the study’s design with a commonly used term in the title or the abstract (b) Provide in the abstract an informative and balanced summary of what was done and what was found | Abstract, Pages 3-4 | RECORD 1.1: The type of data used should be specified in the title or abstract. When possible, the name of the databases used should be included.  RECORD 1.2: If applicable, the geographic region and timeframe within which the study took place should be reported in the title or abstract.  RECORD 1.3: If linkage between databases was conducted for the study, this should be clearly stated in the title or abstract. | Abstract, Page 3  Abstract, Page 3  Abstract, Page 3 |
| **Introduction** | | | | | |
| Background rationale | 2 | Explain the scientific background and rationale for the investigation being reported | Introduction, Pages 5-6 |  |  |
| Objectives | 3 | State specific objectives, including any prespecified hypotheses | Introduction, Page 6 |  |  |
| **Methods** | | | | | |
| Study Design | 4 | Present key elements of study design early in the paper | Methods, Pages 6-10 |  |  |
| Setting | 5 | Describe the setting, locations, and relevant dates, including periods of recruitment, exposure, follow-up, and data collection | Methods, Pages 6-10 |  |  |
| Participants | 6 | *(a) Cohort study* - Give the eligibility criteria, and the sources and methods of selection of participants. Describe methods of follow-up  *Case-control study* - Give the eligibility criteria, and the sources and methods of case ascertainment and control selection. Give the rationale for the choice of cases and controls  *Cross-sectional study* - Give the eligibility criteria, and the sources and methods of selection of participants  *(b) Cohort study* - For matched studies, give matching criteria and number of exposed and unexposed  *Case-control study* - For matched studies, give matching criteria and the number of controls per case | Methods, Page 10  N/A | RECORD 6.1: The methods of study population selection (such as codes or algorithms used to identify subjects) should be listed in detail. If this is not possible, an explanation should be provided.  RECORD 6.2: Any validation studies of the codes or algorithms used to select the population should be referenced. If validation was conducted for this study and not published elsewhere, detailed methods and results should be provided.  RECORD 6.3: If the study involved linkage of databases, consider use of a flow diagram or other graphical display to demonstrate the data linkage process, including the number of individuals with linked data at each stage. | Methods, Page 10  Methods, Pages 8-9, described in text |
| Variables | 7 | Clearly define all outcomes, exposures, predictors, potential confounders, and effect modifiers. Give diagnostic criteria, if applicable. | Methods, Pages 9-10 | RECORD 7.1: A complete list of codes and algorithms used to classify exposures, outcomes, confounders, and effect modifiers should be provided. If these cannot be reported, an explanation should be provided. | The coding of all variables across the EMRs is too voluminous to individually report and provide coding and definitions. |
| Data sources/ measurement | 8 | For each variable of interest, give sources of data and details of methods of assessment (measurement).  Describe comparability of assessment methods if there is more than one group | Methods, Pages 9-10 |  |  |
| Bias | 9 | Describe any efforts to address potential sources of bias | Methods, Page 9-10 |  |  |
| Study size | 10 | Explain how the study size was arrived at | Methods, Pages 8-9 |  |  |
| Quantitative variables | 11 | Explain how quantitative variables were handled in the analyses. If applicable, describe which groupings were chosen, and why | Methods, Pages 9-10 |  |  |
| Statistical methods | 12 | (a) Describe all statistical methods, including those used to control for confounding  (b) Describe any methods used to examine subgroups and interactions  (c) Explain how missing data were addressed  (d) *Cohort study* - If applicable, explain how loss to follow-up was addressed  *Case-control study* - If applicable, explain how matching of cases and controls was addressed  *Cross-sectional study* - If applicable, describe analytical methods taking account of sampling strategy  (e) Describe any sensitivity analyses | Methods, Pages 10-11  Methods, Page 10  Methods, Page 11  N/A |  |  |
| Data access and cleaning methods |  | .. |  | RECORD 12.1: Authors should describe the extent to which the investigators had access to the database population used to create the study population.  RECORD 12.2: Authors should provide information on the data cleaning methods used in the study. | Methods, Pages 9-10  Methods, Pages 9-11 |
| Linkage |  | .. |  | RECORD 12.3: State whether the study included person-level, institutional-level, or other data linkage across two or more databases. The methods of linkage and methods of linkage quality evaluation should be provided. | Methods, Pages 8-9 |
| **Results** | | | | | |
| Participants | 13 | (a) Report the numbers of individuals at each stage of the study (*e.g.*, numbers potentially eligible, examined for eligibility, confirmed eligible, included in the study, completing follow-up, and analysed)  (b) Give reasons for non-participation at each stage.  (c) Consider use of a flow diagram | Methods, Pages 8-9  Methods, Pages 8-9  Supplemental Figure S1 | RECORD 13.1: Describe in detail the selection of the persons included in the study (*i.e.,* study population selection) including filtering based on data quality, data availability and linkage. The selection of included persons can be described in the text and/or by means of the study flow diagram. | Methods, Pages 8-9  Supplemental Figure S1 |
| Descriptive data | 14 | (a) Give characteristics of study participants (*e.g.*, demographic, clinical, social) and information on exposures and potential confounders  (b) Indicate the number of participants with missing data for each variable of interest  (c) *Cohort study* - summarise follow-up time (*e.g.*, average and total amount) | Results, page 12, Supplemental Table 3  Not listed due to extensive number of variables examined  Results, Page 9 |  |  |
| Outcome data | 15 | *Cohort study* - Report numbers of outcome events or summary measures over time  *Case-control study* - Report numbers in each exposure category, or summary measures of exposure  *Cross-sectional study* - Report numbers of outcome events or summary measures | Results, Page 13, Table 1 |  |  |
| Main results | 16 | (a) Give unadjusted estimates and, if applicable, confounder-adjusted estimates and their precision (e.g., 95% confidence interval). Make clear which confounders were adjusted for and why they were included  (b) Report category boundaries when continuous variables were categorized  (c) If relevant, consider translating estimates of relative risk into absolute risk for a meaningful time period | Results, Page 13, Tables 1 and 2  Results, Pages 11-12 |  |  |
| Other analyses | 17 | Report other analyses done—e.g., analyses of subgroups and interactions, and sensitivity analyses | Results, Page 13 |  |  |
| **Discussion** | | | | | |
| Key results | 18 | Summarise key results with reference to study objectives | Discussion, Pages 13-14 |  |  |
| Limitations | 19 | Discuss limitations of the study, taking into account sources of potential bias or imprecision. Discuss both direction and magnitude of any potential bias | Discussion, Pages 15-16 | RECORD 19.1: Discuss the implications of using data that were not created or collected to answer the specific research question(s). Include discussion of misclassification bias, unmeasured confounding, missing data, and changing eligibility over time, as they pertain to the study being reported. | Discussion, Pages 15-16 |
| Interpretation | 20 | Give a cautious overall interpretation of results considering objectives, limitations, multiplicity of analyses, results from similar studies, and other relevant evidence | Discussion, Pages 13-16 |  |  |
| Generalisability | 21 | Discuss the generalisability (external validity) of the study results | Discussion, Pages 15-16 |  |  |
| **Other Information** | | | | | |
| Funding | 22 | Give the source of funding and the role of the funders for the present study and, if applicable, for the original study on which the present article is based | Title Page, Page 1 |  |  |
| Accessibility of protocol, raw data, and programming code |  | .. |  | RECORD 22.1: Authors should provide information on how to access any supplemental information such as the study protocol, raw data, or programming code. | Supplemental information including raw data are not permissible from this analysis. |

*Reference: Benchimol EI, Smeeth L, Guttmann A, Harron K, Moher D, Petersen I, Sørensen HT, von Elm E, Langan SM, the RECORD Working Committee. The REporting of studies Conducted using Observational Routinely-collected health Data (RECORD) Statement. *PLoS Medicine* 2015; in press.

*Checklist is protected under Creative Commons Attribution ([CC BY](http://creativecommons.org/licenses/by/4.0/)) license.

**Supplemental Table 3. Presenting Characteristics of Hospitalized Patients by Admission Wave**

| **Characteristic** | **Wave 1** | **Wave 2** | **Wave 3a** | **Wave 3b** | **Wave 4** | **P-value** |
| --- | --- | --- | --- | --- | --- | --- |
|  | **(N=358)** | **(N=859)** | **(N=3925)** | **(N=4174)** | **(N=2113)** |  |
| Age in years, mean, median | 66.3, 69 | 64.3, 67 | 68.7, 72 | 67.7, 70 | 59.5, 62 | <.001 |
| Age 50 years or younger, (No.), % | (53) 14.8 | (179) 20.8 | (495) 12.6 | (579) 13.9 | (565) 26.7 | <.001 |
| Female gender, (No.), % | (184) 51.4 | (422) 49.1 | (1887) 48.1 | (2004) 48.0 | (1073) 50.8 | .19 |
| Race, (No.), % |  |  |  |  |  |  |
| White | (220) 63.6 | (575) 69.2 | (3284) 86.0 | (3590) 88.2 | (1581) 77.2 | <.001 |
| Black | (122) 35.3 | (233) 28.0 | (467) 12.2 | (433) 10.6 | (453) 22.1 |  |
| Other | (4) 1.2 | (23) 2.8 | (67) 1.8 | (49) 1.2 | (15) 0.7 |  |
| Smoking history, (No.), % |  |  |  |  |  |  |
| Current | (18) 9.6 | (41) 7.4 | (168) 6.3 | (187) 6.7 | (149) 11.3 | <.001 |
| Former | (74) 39.6 | (246) 44.2 | (1247) 46.8 | (1324) 47.4 | (538) 40.8 |  |
| Never | (95) 50.8 | (269) 48.4 | (1250) 46.9 | (1283) 45.9 | (632) 47.9 |  |
| Current alcohol use, (No.), % | (49) 27.2 | (185) 34.3 | (900) 34.6 | (920) 33.7 | (422) 33.5 | .36 |
| Current illicit drug use, (No.), % | (6) 3.4 | (21) 4.0 | (82) 3.2 | (83) 3.1 | (49) 4.0 | .60 |
| **Medical History** |  |  |  |  |  |  |
| History of diabetes, (No.), % | (71) 36.2 | (210) 37.3 | (998) 37.1 | (1053) 37.2 | (429) 32.0 | .01 |
| History of hypertension, (No.), % | (115) 58.7 | (345) 61.3 | (1788) 66.5 | (1852) 65.5 | (830) 61.9 | .004 |
| History of portal hypertension, (No.), % | (33) 16.8 | (72) 12.8 | (334) 12.4 | (332) 11.7 | (154) 11.5 | .24 |
| History of hyperlipidemia, (No.), % | (103) 52.6 | (311) 55.2 | (1605) 59.7 | (1667) 58.9 | (710) 53.0 | <.001 |
| History of morbid obesity, (No.), % | (49) 25.0 | (141) 25.0 | (609) 22.7 | (660) 23.3 | (351) 26.2 | .13 |
| History of obstructive sleep apnea, (No.), % | (40) 20.4 | (126) 22.4 | (594) 22.1 | (636) 22.5 | (272) 20.3 | .56 |
| History of atrial fibrillation, (No.), % | (29) 14.8 | (75) 13.3 | (426) 15.8 | (471) 16.6 | (160) 11.9 | .001 |
| History of coronary artery disease, (No.), % | (45) 23.0 | (117) 20.8 | (697) 25.9 | (739) 26.1 | (274) 20.4 | <.001 |
| History of vascular disease, (No.), % | (13) 6.6 | (46) 8.2 | (243) 9.0 | (255) 9.0 | (105) 7.8 | .51 |
| History of major bleed, (No.), % | (50) 25.5 | (126) 22.4 | (657) 24.4 | (726) 25.7 | (298) 22.2 | 12 |
| History of congestive heart failure, (No.), % | (37) 18.9 | (91) 16.2 | (529) 19.7 | (601) 21.2 | (255) 19.0 | .06 |
| History of asthma, (No.), % | (60) 30.6 | (203) 36.1 | (950) 35.3 | (1032) 36.5 | (500) 37.2 | .38 |
| History of COPD, (No.), % | (40) 20.4 | (132) 23.4 | (658) 24.5 | (738) 26.1 | (350) 26.1 | .21 |
| History of pulmonary embolism, (No.), % | (14) 7.1 | (16) 2.8 | (119) 4.4 | (140) 4.9 | (53) 3.9 | .06 |
| History of chronic kidney disease, (No.), % | (43) 21.9 | (88) 15.6 | (497) 18.5 | (594) 21.0 | (208) 15.5 | <.001 |
| History of cancer, (No.), % | (40) 20.4 | (94) 16.7 | (548) 20.4 | (588) 20.8 | (200) 14.9 | <.001 |
| History of hypothyroidism, (No.), % | (38) 19.4 | (96) 17.1 | (567) 21.1 | (607) 21.5 | (234) 17.4 | .008 |
| History of low back pain, (No.), % | (63) 32.1 | (202) 35.9 | (958) 35.6 | (1000) 35.3 | (439) 32.7 | .34 |
| History of ascites, (No.), % | (50) 25.5 | (115) 20.4 | (719) 26.7 | (723) 25.6 | (277) 20.7 | <.001 |
| History of anxiety, (No.), % | (35) 17.9 | (96) 17.1 | (544) 20.2 | (557) 19.7 | (304) 22.7 | .05 |
| History of depression, (No.), % | (35) 17.9 | (107) 19.0 | (499) 18.6 | (507) 17.9 | (264) 19.7 | .73 |
| CCI total score, mean (median) | 1.8, 1 | 1.5, 1 | 1.7, 1 | 1.7, 1 | 1.5, 1 | <.001 |
| Estimated risk (%) of mortality within 90 days after hospitalization, mean (median) | 29.0, 22 | 22.8, 16 | 27.8, 24 | 26.9, 22 | 20.9, 14 | <.001 |
| **Medications** |  |  |  |  |  |  |
| ACE, (No.), % | (40) 20.4 | (128) 22.7 | (661) 24.6 | (670) 23.7 | (305) 22.7 | .51 |
| ARB, (No.), % | (22) 11.2 | (88) 15.6 | (422) 15.7 | (430) 15.2 | (183) 13.6 | .25 |
| Beta-blocker, (No.), % | (91) 46.4 | (220) 39.1 | (1213) 45.1 | (1279) 45.2 | (506) 37.7 | <.001 |
| Calcium channel blocker, (No.), % | (53) 27.0 | (140) 24.9 | (786) 29.2 | (798) 28.2 | (363) 27.1 | .24 |
| Diuretic, (No.), % | (70) 35.7 | (190) 33.7 | (1022) 38.0 | (1062) 37.5 | (430) 32.1 | .002 |
| DOACS, (No.), % | (33) 16.8 | (66) 11.7 | (399) 14.8 | (411) 14.5 | (147) 11.0 | .003 |
| Warfarin, (No.), % | (13) 6.6 | (29) 5.2 | (177) 6.6 | (184) 6.5 | (49) 3.7 | .002 |
| Anti-platelet, (No.), % | (68) 34.7 | (186) 33.0 | (980) 36.5 | (1007) 35.6 | (375) 28.0 | <.001 |
| Insulin, (No.), % | (43) 21.9 | (115) 20.4 | (504) 18.7 | (499) 17.6 | (205) 15.3 | .02 |
| Metformin, (No.), % | (33) 16.8 | (113) 20.1 | (544) 20.2 | (525) 18.6 | (224) 16.7 | .07 |
| Statin, (No.), % | (109) 55.6 | (295) 52.4 | (1519) 56.5 | (1601) 56.6 | (582) 43.4 | <.001 |
| Short acting bronchodilator, (No.), % | (52) 26.5 | (138) 24.5 | (689) 25.6 | (792) 28.0 | (350) 26.1 | .24 |
| Anti-depressant, (No.), % | (89) 45.4 | (204) 36.2 | (1034) 38.5 | (1102) 39.0 | (459) 34.2 | .005 |
| Corticosteroids, (No.), % | (67) 34.2 | (216) 38.4 | (1097) 40.8 | (1125) 39.8 | (528) 39.4 | .37 |
| Prednisone, (No.), % | (21) 10.7 | (74) 13.1 | (353) 13.1 | (367) 13.0 | (175) 13.1 | .92 |
| Immunomodulators, (No.), % | (4) 2.0 | (7) 1.2 | (62) 2.3 | (71) 2.5 | (23) 1.7 | .26 |
| Muscle relaxant, (No.), % | (19) 9.7 | (64) 11.4 | (316) 11.8 | (300) 10.6 | (168) 12.5 | .36 |
| NSAIDS, (No.), % | (56) 28.6 | (163) 29.0 | (720) 26.8 | (758) 26.8 | (382) 28.5 | .63 |
| Opioids, (No.), % | (42) 21.4 | (147) 26.1 | (709) 26.4 | (757) 26.8 | (314) 23.4 | .10 |
| **Laboratory values** |  |  |  |  |  |  |
| White blood cells, mean, median | 7.6, 6 | 7.3, 6 | 8.1, 7 | 8.6, 7 | 7.6, 7 | <.001 |
| Lymphocytes, mean, median | 15.5, 13 | 16.4, 14 | 14.7, 13 | 13.8, 12 | 15.8, 13 | <.001 |
| Neutrophils, mean, median | 74.6, 76 | 73.1, 75 | 75.1, 78 | 76.2, 78 | 74.3, 77 | <.001 |
| Hemoglobin, mean, median | 12.4, 13 | 12.7, 13 | 12.7, 13 | 12.7, 13 | 13.0, 13 | <.001 |
| Platelets, mean, median | 218.9, 195 | 214.6, 198 | 210.0, 195 | 221.4, 206 | 216.2, 200 | <.001 |
| AST, mean, median | 56.1, 38 | 45.8, 31 | 55.6, 34 | 57.2, 34 | 51.5, 35 | <.001 |
| ALT, mean, median | 42.4, 28 | 37.2, 27 | 42.4, 27 | 45.1, 28 | 42.4, 30 | <.001 |
| Creatinine, mean, median | 1.6, 1 | 1.4, 1 | 1.5, 1 | 1.6, 1 | 1.5, 1 | <.001 |
| Neutrophil to lymphocyte ratio, (No.), % |  |  |  |  |  |  |
| Less than or equal to 3.0 | (56) 21.5 | (150) 22.5 | (633) 19.3 | (568) 16.7 | (360) 21.5 | <.001 |
| 3.0 to 6.0 | (80) 30.7 | (228) 34.2 | (977) 29.7 | (950) 28.0 | (521) 31.1 |  |
| More than 6.0 | (125) 47.9 | (288) 43.2 | (1676) 51.0 | (1876) 55.3 | (795) 47.4 |  |
| Systemic Inflammatory Index, (No.), % |  |  |  |  |  |  |
| SII <=300 | (17) 6.6 | (64) 9.6 | (251) 7.7 | (213) 6.3 | (133) 8.0 | <0.001 |
| SII >300 to <=600 | (48) 18.5 | (133) 20.0 | (558) 17.0 | (457) 13.5 | (293) 17.5 |  |
| SII >600 to <=900 | (40) 15.4 | (103) 15.5 | (477) 14.5 | (456) 13.4 | (288) 17.2 |  |
| SII > 900 | (154) 59.5 | (366) 55.0 | (1997) 60.8 | (2266) 66.8 | (960) 57.3 |  |

Wave 1: - March 19 – June 16, 2020; Wave 2: June 17 – September 19, 2020; Wave 3a: September 20 December 13, 2020; Wave 3b: December 14, 2020 – March 10, 2021; Wave 4: March 11 – June 6, 2021.
