# Supplementary figures and images for: A learning health system approach to the COVID‐19 pandemic: System‐wide changes in clinical practice and 30‐day mortality among hospitalized patients
Source: Learn Health Syst. 2022 Jan 27;6(3):e10304. doi: 10.1002/lrh2.10304 (PMC9284933; doi:10.1002/lrh2.10304)

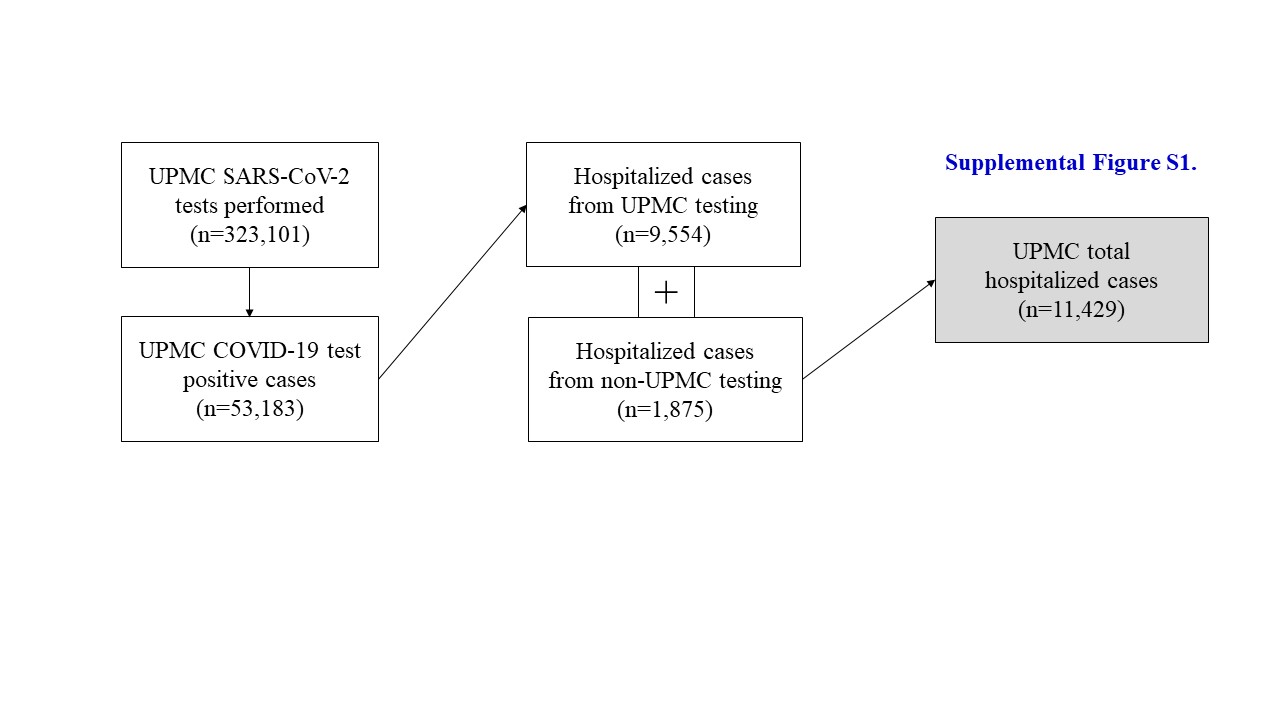

Supplement: Supplementary file 2 — Figure S1 Diagram of severe acute respiratory syndrome coronavirus 2 (SARS‐CoV‐2) testing performed within the University of Pittsburgh Medical Center (UPMC) system, including COVID‐19 hospitalized patients aggregated from patients tested within and outside of the UPMC system. [file LRH2-6-e10304-s003.jpg]

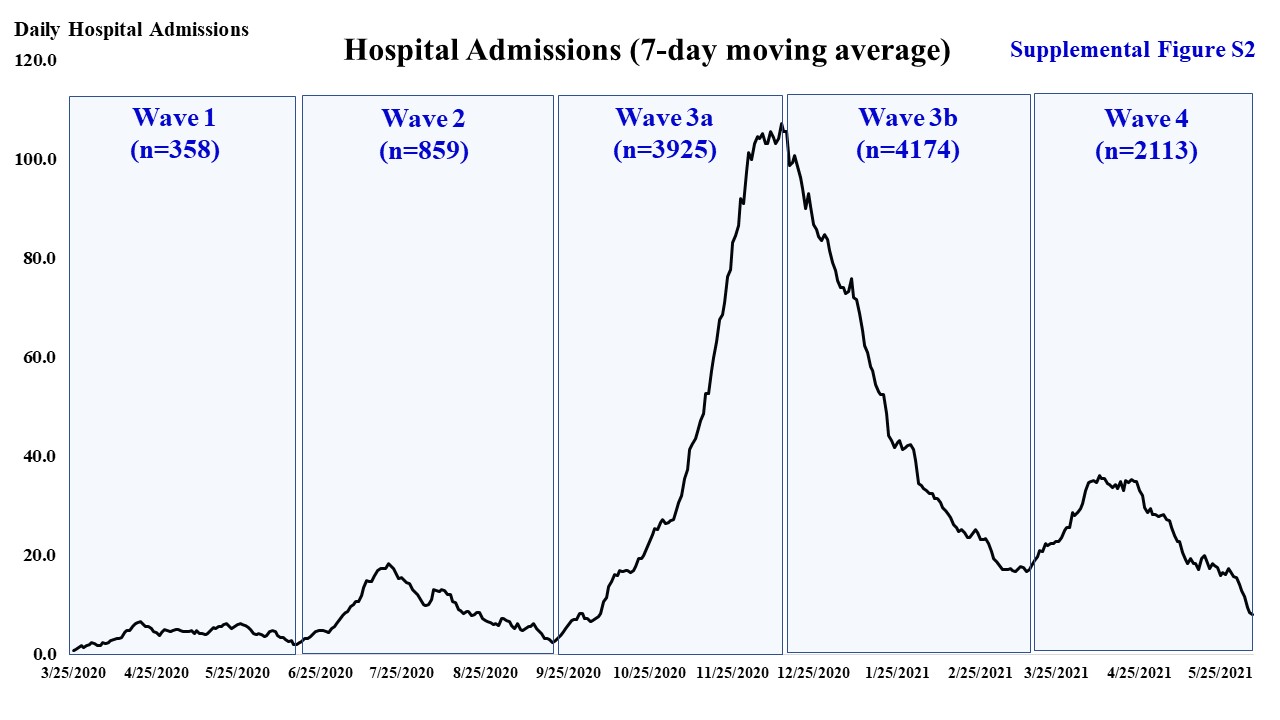

Supplement: Supplementary file 3 — Figure S2 Plot of 7‐day moving average of coronavirus disease 2019 (COVID‐19) hospital admissions by empirically defined “waves” based on nadir and zenith (wave 3) of admissions. The time periods for the waves were as follows: Wave 1: ‐ March 19 ‐ June 16, 2020; wave 2: June 17 ‐ September 19, 2020; wave 3a: September 20‐December 13, 2020; wave 3b: December 14, 2020 ‐ March 10, 2021 and wave 4: March 11 ‐ June 6, 2021. [file LRH2-6-e10304-s007.jpg]

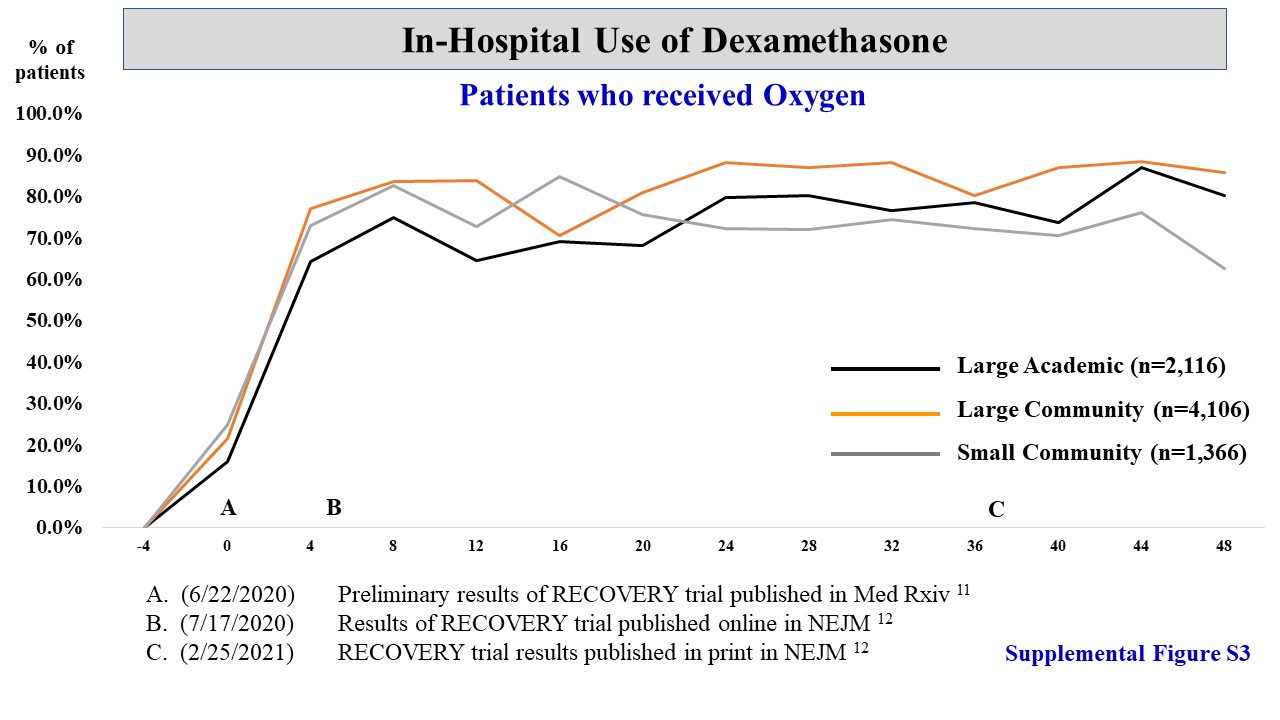

Supplement: Supplementary file 4 — Figure S3 Plot of 4‐week prevalence (%) of in‐hospital use of dexamethasone among patients who received oxygen by hospital classification. On the x‐axis, negative numbers reflect weeks prior to seminal event “A”, the date (June 22, 2020) in which preliminary results of the RECOVERY trial were published in Med Rxiv. Positive numbers reflect weeks after seminal event A. [file LRH2-6-e10304-s004.jpg]

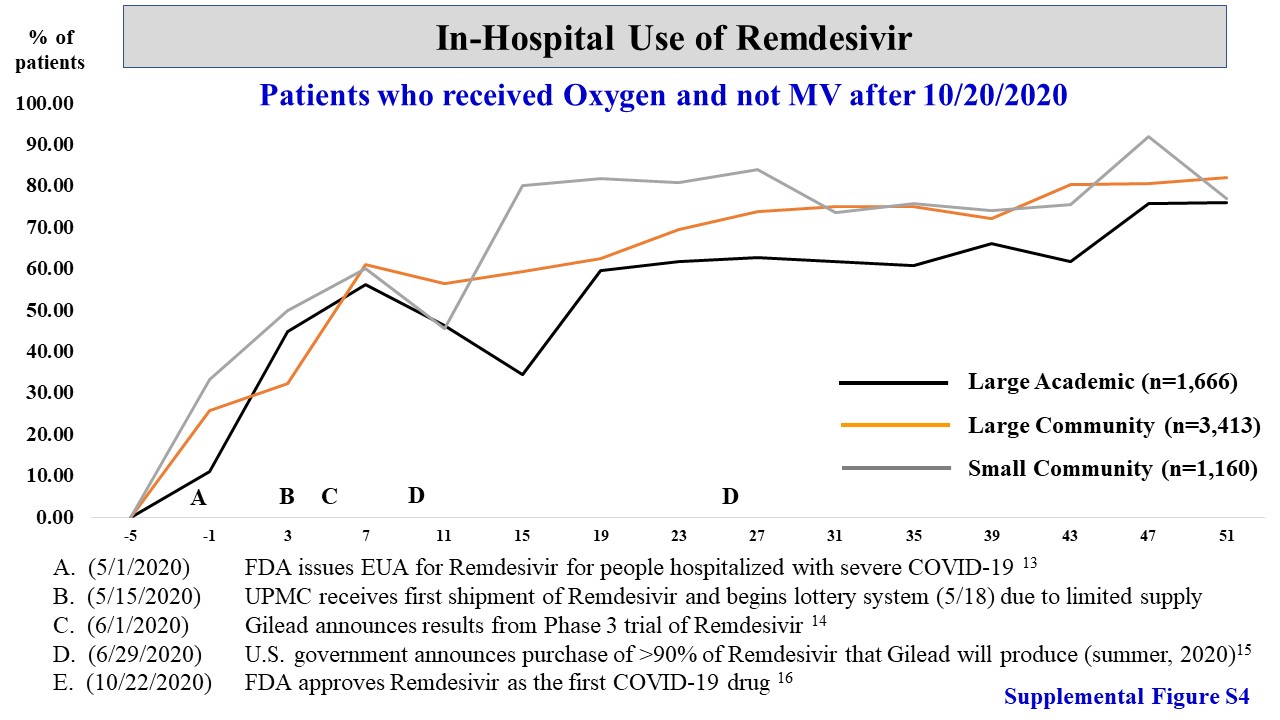

Supplement: Supplementary file 5 — Figure S4 Plot of 4‐week prevalence (%) of in‐hospital use of remdesivir among patients who received oxygen (but not mechanical ventilation after October 20, 2020) by hospital classification. On the x‐axis, negative numbers reflect weeks prior to seminal event “A,” the date (May 1, 2020) in which the Food and Drug Administration (FDA) issued Emergency Use Authorization (EUA) for remdesivir for patients hospitalized with severe coronavirus disease 2019 (COVID‐19). [file LRH2-6-e10304-s001.jpg]

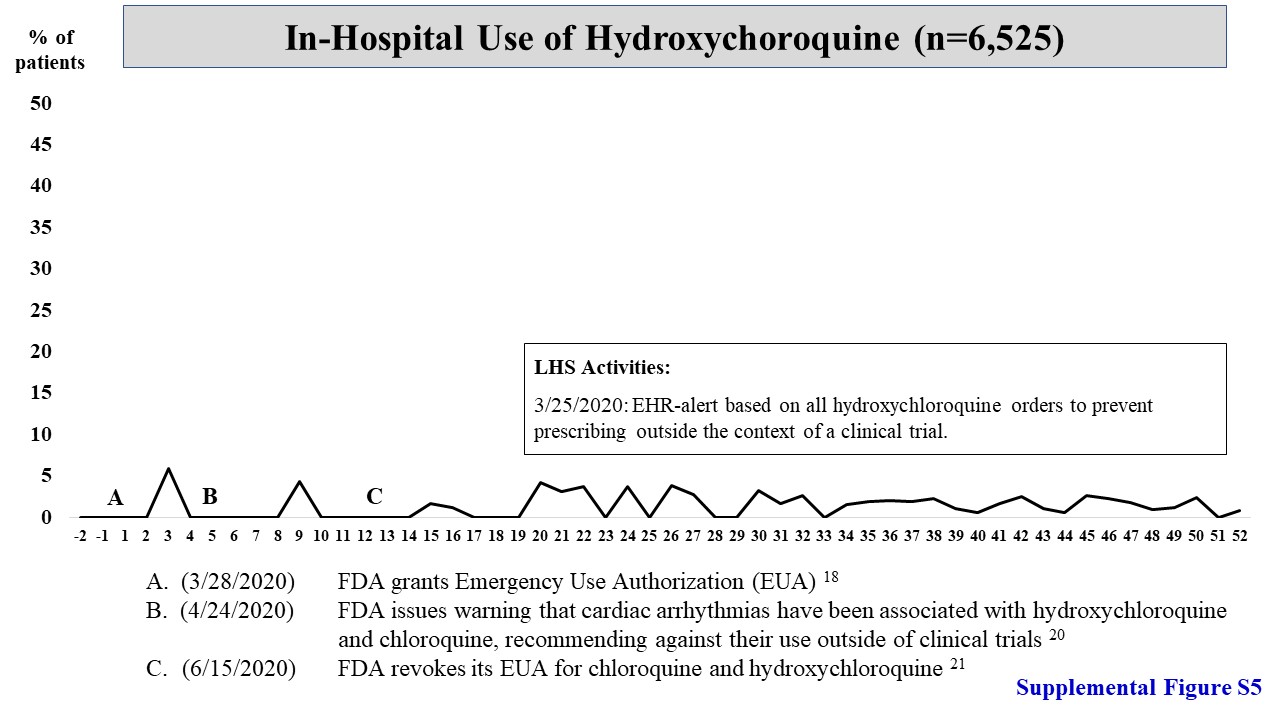

Supplement: Supplementary file 6 — Figure S5 Plot of weekly prevalence (%) of in‐hospital use of hydroxychloroquine. On the x‐axis, negative numbers reflect weeks prior to seminal event “A,” the date (March 28, 2020) in which the Food and Drug Administration (FDA) granted EUA of hydroxychloroquine for coronavirus disease 2019 (COVID‐19) patients. [file LRH2-6-e10304-s002.jpg]

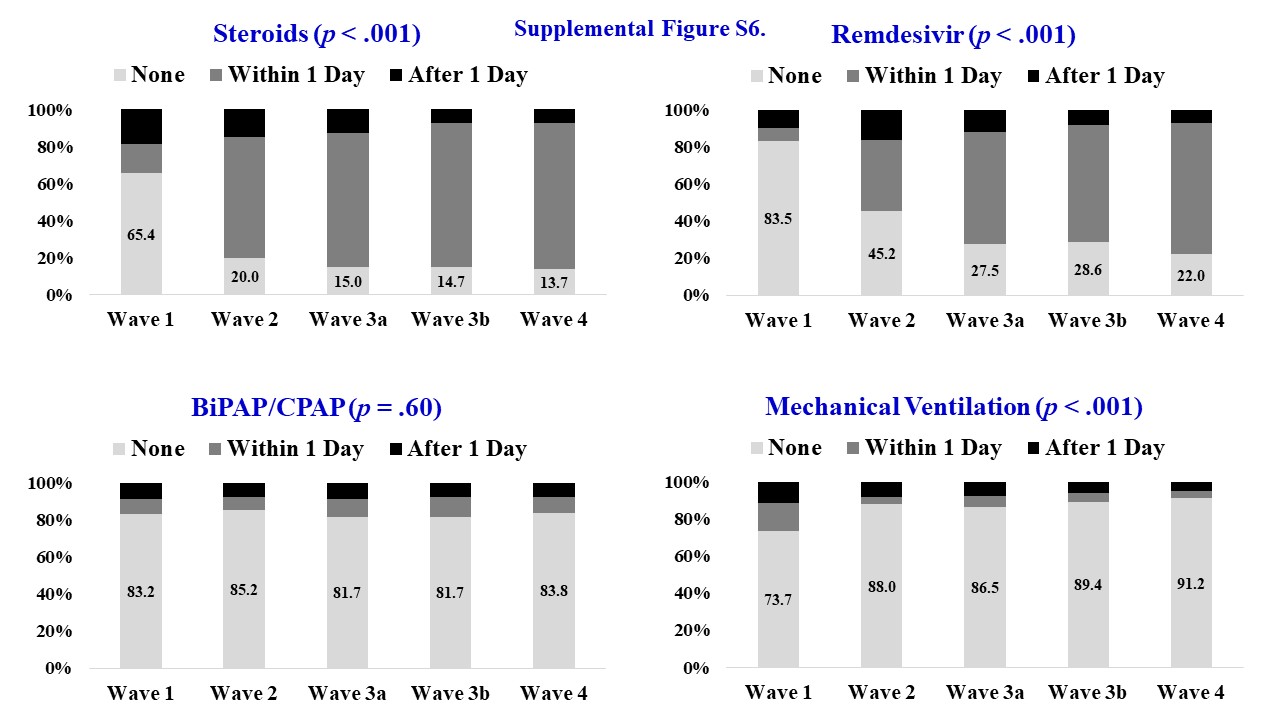

Supplement: Supplementary file 7 — Figure S6 Stacked bar charts (100%) of the percentage of hospitalized patients treated with steroids (upper left), remdesivir (upper right), bilevel positive airway pressure (BiPAP)/continuous positive airway pressure (CPAP) (lower left), and mechanical ventilation (lower right) by wave of hospital admission. For steroids, the denominator is patients on oxygen therapy. For remdesivir, the denominator is patients on oxygen therapy and not mechanical ventilation after October 20, 2020. Light shading: treatment not used; intermediate shading: treated provided within 1 day of hospital admission; darker shading: treatment provided after the first day of hospital admission. P‐values are based on the Cochran‐Mantel‐Haenszel test of trend. [file LRH2-6-e10304-s006.jpg]
